# *OsPAL2-1* Mediates Allelopathic Interactions Between Rice and Specific Microorganisms in the Rhizosphere Ecosystem

**DOI:** 10.3389/fmicb.2020.01411

**Published:** 2020-07-23

**Authors:** Yingzhe Li, Xin Jian, Yue Li, Xiaomei Zeng, Lining Xu, Muhammad Umar Khan, Wenxiong Lin

**Affiliations:** ^1^Fujian Provincial Key Laboratory of Agroecological Processing and Safety Monitoring, College of Life Sciences, Fujian Agriculture and Forestry University, Fuzhou, China; ^2^Key Laboratory of Crop Ecology and Molecular Physiology (Fujian Agriculture and Forestry University), Fujian Province University, Fuzhou, China; ^3^Key Laboratory of Ministry of Education for Genetics, Breeding and Multiple Utilization of Crops, College of Agriculture, Fujian Agriculture and Forestry University, Fuzhou, China

**Keywords:** allelochemicals, allelopathic interaction, *Myxococcus xanthus*, rice, phenolic acid

## Abstract

The use of plant allelopathy to control weeds in the field has been generally recognized as a win-win strategy because it is an environmentally friendly and resource-saving method. The mechanism of this natural weed-control method relies on allelochemicals, the rhizosphere microbiome, and their bio-interaction, and exploring the link between allelochemicals and specific microbes helps accelerate the application of allelopathic characteristics in farming. In this study, we used allelopathic rice PI312777 (PI), its genetically modified *OsPAL2-1* repression (PR) or overexpression (PO) lines, and non-allelopathic rice Lemont (Le) as donor plants to reveal the bio-interaction between rice allelochemicals and rhizosphere specific microorganisms. The results showed a higher content of phenolic acid exudation from the roots of PI than those of Le, which resulted in a significantly increased population of *Myxococcus* in the rhizosphere soil. Transgenic PO lines exhibited increasing exudation of phenolic acid, which led to the population of *Myxococcus xanthus* in the rhizosphere soil of PO to be significantly increased, while PR showed the opposite result in comparison with wild type PI. Exogenous application of phenolic acid induced the growth of *M. xanthus*, and the expressions of chemotaxis-related genes were up-regulated in *M. xanthus*. In addition, quercetin was identified in the culture medium; according to the bioassay determination, a quercetin concentration of 0.53 mM inhibited the root length by 60.59%. Our study indicates that *OsPAL2-1* is among the efficient genes that regulate rice allelopathy by controlling the synthesis of phenolic acid allelochemicals, and phenolic acid (ferulic acid, FA) induces the chemotactic aggregation of *M. xanthus*, which promoted the proliferation and aggregation of this microbe. The potential allelochemical, quercetin was generated from the FA-induced *M. xanthus* cultured medium.

## Introduction

Plant allelopathy is an ecological phenomenon involving the interaction of multiple factors including plants, microorganisms, allelochemicals, and the environment. There are two main types of allelochemicals in rice. Phenolic acids are considered to be one group of allelochemicals, and the other group contains terpenoids and flavonoids. Chou et al. (1981); Rice (1984) and Chou, 1989 believed that phenolic acids, such as *p*-coumaric acid, ferulic acid, *p*-hydroxybenzoic acid, and oxalic acid, produced during the decomposition of rice residues, could be fixed by soil aggregate structural substances or humic acid and stored in the rhizosphere soil to inhibit the growth of rice seedlings and weeds (Chou et al., 1981; Rice, 1984; Chou, 1989; Rimando et al., 2001; Seal et al., 2004). However, Olofsdotter et al. (2002) had a skeptical view of this issue, suggesting that phenolic acid compounds might not be the allelochemicals that inhibit target weeds, since she considered that 4-aminoantipyrine spectrophotometry, often used for the determination of the action dosages, was unsuitable. Moreover, there are documents that indicate that it is not effective unless 4-aminoantipyrine spectrophotometry is used to determine the contents of non-volatile phenolic substances in root exudates from rice (Li et al., 2017). He et al. (2004) used an orthogonal regression design to determine the allelopathic action under laboratory conditions and found that the optimal mixture of five phenolic acids had a higher inhibition rate than each of the individual phenolic acids, which aided in the suppression of barnyard grass. This result suggested that the allelopathic potential of rice might not be related to the concentration of a single allelochemical but rather to the synergistic effect of multiple phenolic acid allelochemicals in an optimal mixture, which explained in part why each of the phenolic acids had a higher inhibitory effect on the target weeds in the bioassay under laboratory conditions but not in the field, simply because the action dosage was much higher in the laboratory than in the field. Terpenoids, such as momilactone B, are often considered promising allelochemicals of rice, since they have a higher inhibitory effect on target weeds at low dosages of 3–30 μM compared with phenolic acids (Kato-Noguchi, 2004, 2011; Kato-Noguchi and Ino, 2005; Wang et al., 2010; He et al., 2012).

However, all of the aforementioned results were obtained under laboratory conditions. Moreover, the effective concentrations of phenolic acids and terpenoids used for laboratory bioassays were all higher than those detected in allelopathic rice under field conditions, which therefore has frequently been questioned by some scholars. With the development of research work, scientists have gradually understood that allelopathy is a quantitative trait, which is involved in an extremely complex chemobiological process in the rhizosphere ecosystem. Shin et al. (2000) found that the genes coding the two key enzymes, participating in the pathway of phenolic acid synthesis, cinnamate 4-hydroxylase (CA4H) and phenylalanine ammonia-lyase (PAL), were upregulated earlier and to a higher level. Consequently, the encoded enzymic activities and the products of phenolic acid synthesis in the pathway were also higher in allelopathic rice than in its counterpart non-allelopathic rice when the two rice accessions were exposed to U-VB treatment (Shin et al., 2000). It was also found that the allelopathic potential of rice was significantly enhanced with the increase in barnyard grass density in a coculture treatment under hydroponic conditions (He et al., 2012). Further studies by our research group also showed that the expression of all genes coding for nine enzymes associated with the phenolic metabolism pathway was up-regulated in allelopathic rice PI312227 under low nitrogen stressful conditions compared with a normal nitrogen supply using a qPCR (real-time quantitative PCR detecting system) approach (Xiong et al., 2007). The reverse was true in the case of a non-allelopathic rice accession under the same nitrogen condition, except that the two genes coding for cinnamate CoA ligase and phenylalanine ammonia-lyase showed a slight up-regulation in non-allelopathic rice than in allelopathic rice under the nitrogen deficiency condition (Xiong et al., 2007). Therefore, it was suggested that the enhanced weed inhibition ability of allelopathic rice was mainly related to the significant up-regulation of some gene members of the *PAL* family. Based on our previous research work on the differential expression patterns of 11 members of the *PAL* family genes in rice roots with different allelopathic potential under different types of environmental stress, we found that most of the *PAL* family genes in allelopathic rice accessions were up-regulated in response to nitrogen deficiency or a high accompanying density of barnyard grass, and the enhanced expression of the *PAL-2-1* gene was the most obvious among the gene members of the *PAL* family. However, the differential expression abundance of the *PAL* family genes, especially *PAL-2-1* genes in non-allelopathic rice, was not significant under the same treatment, implying that the *PAL-2-1* gene might be related to the regulation of the enhanced chemical defense ability of allelopathic rice in early response to environmental stress (Fang et al., 2011). Further studies confirmed that the phenolic acid synthesis was blocked in the *PAL-2-1-* inhibited transgenic line of allelopathic rice PI312777, and subsequently, the ability to inhibit barnyard grass was significantly decreased, indicating that the *PAL-2-1* gene is one of the functional genes closely related to the ability of allelopathic rice to inhibit grass (Fang et al., 2013). More recently, in order to further explain why a lower action dosage of phenolic acids detected in rhizosphere soil is able to produce a higher allelopathic effect on the target weeds under field conditions, further studies have been conducted, and the results suggested that plant allelopathy includes direct allelopathic effects caused by allelochemicals, and indirect allelopathic effects mediated by allelochemicals through microbial utilization, transformation, and resynthesis. Therefore, we speculated that allelopathy in rice might result from the interaction of allelochemicals with specific microorganisms, such as myxobacteria in rhizosphere soil (Vivanco et al., 2004; Lin et al., 2007; Kong et al., 2008; Qu and Wang, 2008), which needs further investigation (Xiong et al., 2012; Fang et al., 2013, 2015; Lin, 2013).

Our previous research comparatively studied the composition difference of microbial communities in the rhizosphere soil of allelopathic and non-allelopathic rice accessions and found 31 unique microbial genera in the rhizosphere soil of allelopathic rice PI312777, seven of which belongs to myxobacteria at the rice seedling stage (3–7 leaf stage) (Xiong et al., 2012). Fang et al. (2015) documented that the inhibitory effect of allelopathic rice accessions on target barnyard grass under field conditions might be related to miRNA expression, which is associated with nucleotide excision repair, auxin signal and its transduction pathway in target barnyard grass, thereby reducing its ability to repair DNA damage, subsequently resulting in blocked synthesis of auxin and ultimately repressing the growth of barnyard grass. However, what is involved in the rhizosphere biological process still remains unknown. We speculated that the underlying mechanism might be associated with the process through the chemotaxis and mediation of specific myxobacteria by phenolic acid secretion from the roots of allelopathic rice accessions in the rhizosphere ecosystem. Recent research work has suggested that plant root exudates, such as low-molecular-weight organic compounds, amino acids, and plant secondary metabolites, could effectively shift the structure and function of soil microbial community and thus affect the interaction between plants and microorganisms (Bressan et al., 2009; Shi et al., 2011), implying that rice allelopathy might result from the interaction between the specific myxobacteria and allelochemicals in rhizosphere soil ecosystems.

Myxobacteria have the largest genome among prokaryotes, giving them a large number of luxury genes (Weissman and Müller, 2009). These not only make myxobacteria have complex multicellular behavior but also allow them to produce rich secondary metabolites that have a wide range of anti-eukaryotic biological activities (Weissman and Müller, 2009). The multicellular population behavior of myxobacteria is regulated by complex signal networks, and group life is realized through unique sliding movement, namely the social motility system (S-motility system) and adventure motility system (A-motility system) (Zusman et al., 2007). The completion of the S movement requires cell-to-cell cooperation (Mauriello et al., 2010), which is controlled by the Frz allelopathic system (Sun et al., 2000). Early research found that the chemotactic gene family coding for Frz of myxobacteria contains seven gene members: *FrzA, B, CD, E, G, F*, and *Z* (Shi and Zusman, 1995), which are homologous with the chemotactic gene of *Escherichia coli*. It was documented that *FrzA* and *CheW* have functional similarity. The same was true in the case of *FrzE*, *CheA*, and *CheY* (Zusman and McBride, 1991). In addition, in the presence of ATP, *FrzE* is able to be autophosphorylate (McBride et al., 1993); *FrzF* and *FrzG* play the same role of *CheR* and *CheB* in chemotaxis. *FrzCD* is a methyl-accepting chemotaxis protein (MCP). Methylated *FrzCD* is related to how cells perceive nutrients or substances in their environment (Dworkin, 1996). Only the FrzB protein is unique to myxobacteria. Further studies revealed the chemotactic mechanism of *Myxococcus xanthus*, such as *CheC* and *CheD*, which act similarly to a chemotactic two-component signal transduction system (Kirby and Zusman, 2003). Further studies found that the Frz system consists of eight genes, namely, *FrzA, FrzB, FrzCD, FrzE, FrzF, FrzG, FrzS*, and *FrzZ*, which are related to differentiation and movement, which are involved in the predation behavior of myxobacteria (Pham et al., 2005). *M. xanthus* is often used as a model strain for the study of multicellular behavior (Hans, 1999), and this specific microbe strongly relies on a signal transduction pathway to regulate its own cellular behavior and complete the quorum sensing reaction in some specific bacteria, and at least five signal factors, such as A (Asg), B (Bsg), C (Csg), D (Dsg), and E (Esg), have been determined (Downard et al., 1993), of which C signal encoded by *csg*A, works at the last time of all signals, and plays an important role in cell aggregation and spore formation during the aerobic growth of *M. xanthus*. The signal is transduced only when cells are in contact with each other, and this in turn results in the expression of genes related to cell aggregation and movement (Søgaard-Andersen et al., 1996; Kaiser, 2003). The expressed C signal further activates the FruA protein through post-transcriptional modification, and the FruA protein is necessary for the processes of cell aggregation and sporulation, which requires both the A signal and E signal for its synthesis. *FruA* (Ellehauge et al., 1998) has a target, the *Frz* gene, which is equivalent to a phosphate repeater. The transcribed activated FruA protein transmits the signal along the frz phosphate repeater, methylating the FrzCD protein; as a result, the fruiting body is formed in this process. Therefore, rice allelopathy is a very complex process of rhizosphere biology, and we need to further uncover the underlying mechanism involved in the specific microbe and allelochemicals in soil ecosystems.

In order to further investigate the correlation between rice *OsPAL2-1* gene expression abundance and myxobacteria populations in its rhizosphere the possible link between them needs to be explored. In this study, allelopathic rice PI312777 (PI), its genetically modified *OsPAL2-1* repression (PR) or overexpression (PO) lines, and non-allelopathic rice Lemont (Le) were taken as research materials; these genetically modified lines were developed in our previous studies (Fang et al., 2015; Li et al., 2020). The population number of *Myxococcus* sp. from the rhizosphere of these rice and the phenolics contents in the rice were detected. *Myxococcus xanthus* from *Myxococcus* sp. was isolated from rice rhizosphere soil to investigate the bio-interaction with phenolics, and to evaluate the inhibitory effect of *M. xanthus* and the metabolites on the growth of barnyard grass. The subsequent studies attempted to further elucidate the mechanism and mode of action of allelochemicals on specific microorganisms colonizing in the rhizosphere soil of allelopathic rice so as to provide a theoretical basis for further understanding the biochemical process and mechanism of rice allelopathy in the suppression of target weeds in rhizosphere soil ecosystems.

## Materials and Methods

### Preparation of Strain and Plant Materials

#### Rice Accessions Used in the Experiment

The following materials were used: allelopathic rice accession PI312777 (abbreviated as PI), and its genetic derivatives, of which the gene expression of *OsPAL2-1* was inhibited or overexpressed, according to the technique of RNA interference (PAL-RNAi, PR) or overexpression (PAL-overexpression, PO), respectively, and non-allelopathic rice Lemont (Le) developed and provided by the Institute of Agroecology, Fujian Agriculture and Forestry University, Fuzhou, China (Fang et al., 2013; Li et al., 2020) were used as the test materials. *OsPAL2-1* is known as the key gene involved in the regulation of the phenylalanine metabolism pathway, and the expression level of *OsPAL2-1* determines the contents of the phenolic acids in rice. CK refers to the blank control. The seeds of all the test rice were sterilized using sodium hypochlorite for 30 min, and soaked in sterilized ddH_2_O for 24 h, and then soaked in hygromycin (50 mg/mL) for 24 h. After that, the hygromycin was completely removed, and the seeds were pre-germinated in petri dishes in a 30°C incubator for 2–3 days. The experiment was conducted under field conditions and in the indoor laboratory at the Institute of Agroecology, Fujian Agriculture and Forestry University, from March 2017 to September 2018.

During the experiment, the annual average temperature was 20–25°C, the annual average maximum temperature in the daytime was 26°C, and the annual average minimum temperature at night was 16°C. The average monthly temperature during the early rice planting month from March to April was between 20°C and 26°C, and the average temperature of the late rice planting month from July to September ranged from 33 to 37°C. The annual relative humidity was about 77%.

#### Field Experimental Design and Soil Sample Collection

The test seedlings of all rice entries were cultured in a dry-raised nursery followed by the method described by Li et al. (2018). The field soil contained 1.94 g⋅kg^–1^ total nitrogen (potassium dichromate-sulfuric acid digestion method), 61.37 mg⋅kg^–1^ alkali hydrolyzable nitrogen (alkali hydrolysis diffusion method), 0.76 g⋅kg^–1^ total phosphorus (sulfuric acid-perchloric acid digestion method), 30.7 mg⋅kg^–1^ available phosphorus (phosphomolybdate blue colorimetry NH_4_F-HCl extraction), 1.68 g⋅kg^–1^ total potassium (NaOH melting-flame photometer method), 208.64 mg⋅kg^–1^ available potassium (flame photometry after extraction with 1M ammonium acetate solution buffered at pH 7), and 22.5 g⋅kg^–1^ organic matter (potassium dichromate volumetric method), with a pH value of 6.21 (a portable pH meter, IQ 150, Spectrum technologies Inc., Aurora, IL, United States) in the tillage layer (Bao, 2000; Pansu and Gautheyrou, 2007). Rice seedlings were transplanted on 25 March 2017 and 25 July 2018, with appropriate basal fertilizers (70% of total N as basal dressing and the rest as top dressing, with total *N* = 225.0 kg/ha, *P* = 29.5 kg/ha, *K* = 149.4 kg/ha). The three nutrient elements N, P, and K were applied in the form of urea (N), calcium superphosphate (P), and potassium chloride (K), respectively, to each plot according to the proportion of the three elements as mentioned above. Each variety was planted separately in different plots (3 m × 1 m), and this area was evenly divided into three parts (1 m × 1 m); the seeding rate was 150 g/m^2^. In addition, CK was set as the blank control soil in the same area without rice. After the emergence of rice seedlings, field management followed recommended methods. Random sampling was conducted in each treatment at the 3-, 5-, and 7-leaf stages of rice seedlings.

#### Pot Trials

Pot trials were carried out in a plastic barrel (outer diameter of 33 cm and inner diameter of 31 cm), 12.0–12.5 kg, per barrel of sun-dried soil, and an appropriate amount of fertilizer per bucket (converted according to *N* = 225 kg/ha, *P* = 29.5 kg/ha, *K* = 149.4 kg/ha, applied in the form of urea, calcium superphosphate and potassium chloride, conversion according to barrel area, that is 0.64, 2.81, 0.45 grams of urea, calcium superphosphate and potassium chloride basal dressing, and 0.183 and 0.091 grams of urea for topdressing per bucket, respectively), and irrigated with 3 liters of water. Pre-germinated seeds were seeded on 21 April 2017, at 15 seeds per barrel. CK (blank control without rice), and the PI, Le, PR, and PO lines were directly seeded in 10 barrels. When the rice grew to the 1-leaf stage, five rice uniform plants were retained per barrel. The same amount (1 L) of tap water was applied every day, and topdressing was applied twice during the seedling stage at 3- and 5-leaf stages. Potted plants were placed in a growth chamber and were sheltered from rain with white plastic. Temperature varied from 25 to 30°C, while humidity varied from 65 to 78% during the experiment. Random soil sampling was conducted at the 3-, 5-, and 7-leaf stages of the rice seedlings.

#### The Collection of Blank Soil and Rhizosphere Soil Samples

From the plots without rice, surface soil was removed along with vegetation to collect the 0–5 cm deep soil layer. The uniform rice plants grown in pots were carefully uprooted preventing any damage to the roots. The soil sticking to the rhizoplane was brushed off and collected as rhizosphere soil samples. All of the soil samples were stored in a refrigerator until needed for tests.

### Isolation and Identification of the Key Microorganism, *Myxococcus xanthus*, in the Rhizosphere Soil of Allelopathic Rice

The rabbit feces induction method (Zhang et al., 2003) was used in this experiment. The procedure is as follows: First, 10 g of the rhizosphere soil of PI was air-dried and placed in a tissue culture bottle with 20 mL of sterilized ddH_2_O. Then, a filtered and sterilized mixed solution of antibiotics (gentamicin 40 mg/mL, kanamycin 10 mg/mL, and ampicillin 40 mg/mL, total 1 mL, and actinomycetoma 40 mg/mL dissolved in 1 mL dimethyl sulfoxide solution) was added to the tissue culture bottle. The culture bottle was placed in a water bath at 55°C for 10 min and then stored overnight at room temperature. Second, rabbit feces were collected and sterilized and then stored in tissue culture bottles. Third, fruiting bodies were induced by the specific microbe: The excess liquid from the treated soil sample was poured out, and the wet soil (approximately 3 mm thick) was placed on a sterile plate. Four or five pieces of sterilized rabbit feces were half-buried in the treated soil sample with tweezers and cultured at 28°C. After 48 h, the formation of fruiting bodies on the rabbit feces could be observed. Then, strains were screened and purified: the induced fruiting bodies were picked out on sterilized VY/4 medium (0.5% yeast powder; CaCl_2_⋅2H_2_O 0.2%, agar 2%, pH 7.2) with an inoculation ring for subgeneration and subcultured at 28°C. A large number of vegetative cells could be observed within 24 h, and fruiting bodies of the microbe were formed within 48 h. After repeated subculture until there were no other miscellaneous bacteria, the expected *Myxococcus* strains were screened, proliferated and preserved. Finally, the strain was preserved at normal culture temperature in the following way: The bacteria were cultured on VY/4 medium and were stored at 28°C after colony growth. The new colonies were marked, purified, and preserved at regular intervals. The bacterial cryopreservation for subsequent studies was as follows: 600 mL bacteria liquid was placed in a 1.5 mL EP tube; 60% glycerol of the same volume was added followed by storage at −80°C.

#### DNA Extraction of the Isolated Strain

Due to the influence of the unique biological characteristics of myxobacteria, the traditional method for the extraction of bacterial DNA was not ideal, the amount of extracted DNA was low and impurities were obtained. After considerable exploration, we referred to the extraction method that used a small amount of DNA (Kutchma et al., 1998) and the CTAB/NaCl precipitation method of bacteria, and in a short time, we achieved large amounts of highly pure extracted DNA, through the combination and simplification of the two methods.

The specific methods are as follows. First, the isolated and purified strain was picked up in VY/4 liquid medium and cultured for 72 h at 28°C, 150 rpm. Second, the mature bacterial liquid was centrifuged and suspended in 750-μL DNA extract liquid (0.25 M EDTA (pH = 8.0), 0.1 M Tris–HCl (pH = 8.0), 2% CTAB, 2% PVP, 2 M NaCl_2_, 2 M CaCl_2_, 0.4 M LiCl), and then placed in a water bath at 55°C for 10 min. Subsequently, the same volume of Tris equilibrium phenol-chloroform-isoamyl alcohol (25:2:1) was added, mixed well (shaken), and then centrifuged to obtain the supernatant. Depending on the purity of the bacterial solution, the DNA was repeatedly extracted two to three times until there was no protein layer. Then, the same volume of isopropanol was added to the supernatant and passed through the DNA recovery column followed by a 70% ethanol washing column; this step was repeated followed by blow drying. Finally, DNA was eluted from the column with a suitable amount of sterile water.

#### PCR Verification and Identification of the Strain

After the total DNA extracted by the above method was recovered by a DNA Gel Extraction Kit (Omega, United States), the 16S rRNA gene sequence was amplified by bacterial universal primers (P1:5′-AGAGTTTGATCCTTGGCTCAG-3′ and P2:5′-AGAAAGGAGGTGATCCAGCC-3′). The PCR system consisted of 25 μL as follows: template 1 μL, primer 1 1 μL, primer 2 1 μL, 2 × EcoTaq PCR Supermix 12.5 μL, H_2_O 9.5 μL. The amplification conditions were as follows: 95°C for 5 min, 95°C for 1 min, 55°C for 50 s, 72°C for 90 s, and 72°C for 10 min. The PCR products were purified using a UNIQ-10 spin column DNA Gel Extraction Kit (Shanghai Sangon Biotech), and the samples were sent to the Shanghai Sangon Biotech Sequencing Center for DNA sequencing. The sequence was then alignment in GenBank using Blastn.

### The Interaction of *Myxococcus xanthus* With Allelopathic Rice Accessions

#### qPCR Analysis of *M. xanthus* in the Rhizosphere Soil of Allelopathic Rice Accessions

The total DNA of the CK, PI, PR, and PO rhizosphere soil sampled from the pot trials was extracted in four replicates according to Peršoh et al. (2008). The absolute fluorescence quantitative PCR (qRT-PCR) of *Myxococcus* spp. in rhizosphere soil was performed following Martin and Bull (2006) using nested PCR. The first round of amplification was a common PCR. The primers were Myxo70F, 5′-CGCGAATAGG-GGCAAC-3′, and Myxo1346R, 5′-GCAGCGTGCTGATCTG-3′. The PCR procedure was as follows: pre-denaturation at 95°C for 5 min, 95°C for 30 min, 58°C for 1 min, 72°C for 1 min, for 25 cycles, and extension of 5 min at 72°C. The PCR products were performed by electrophoresis and stored in a refrigerator at 4°C for the second round of PCR. The second round of quantitative real-time PCR fluorescence analysis was performed on an Eppendorf realplex4 quantitative PCR instrument. The primers were Myxo184F, 5′-CACG-GTTTCTTCGGAGACT-3′, and Myxo1128R, 5′-CTCTAGA-GATCCACTTGCGTG-3′. The configuration was based on the SYBR Premix Ex TaqTM (TaKaRa, China) instruction manual, and the reaction system was 15 μL. The reaction parameters were as follows: pre-denaturation at 95°C for 15 min, denaturation at 95°C for 20 s, annealing at 61°C for 20 s, extension at 72°C for 40 s, for 41 cycles, and then 95°C for 15 s, 60°C for 15 s, heating for 10 min to 95°C (reading fluorescence once every 0.2 degrees), and then 95°C for 15 s to form a dissolution curve. According to the threshold (CT value) generated by the software, the 2-ΔΔct method was used to calculate the relative gene expression. Each DNA sample of five rice accessions in rhizosphere soil was replicated four times.

#### Induction Effects of *M. xanthus* by Different Concentrations of Phenolic Acid

Optimal concentrations of ferulic acid, cinnamic acid, vanillic acid, and *p*-hydroxybenzoic acid were prepared following the method described by He et al. (2004), and these phenolic acids were, respectively, added to 1/2 CTT medium, to investigate the effect on the *M. xanthus*. Then, dynamic sampling was carried out, and the growth curve was drawn by the weighing method. Six replicates were used for each treatment, and the treated concentration of each phenolic acid was prepared and used as shown in Table 1.

**TABLE 1:** The types and treated concentration levels of each phenolic acid inducing the growth of *Myxococcus xanthus.*

Factor	Concentration levels (mmol⋅L^–1^)
*p*-hydroxybenzoic acid	0.40 0.50 0.60
Cinnamic acid	0.12 0.20 0.28
Vanillic acid	0.02 0.10 0.18
Ferulic acid	0.05 0.1 0.15

#### Inoculation Effect of Mixed Phenolic Acids on *M. xanthus* in Soil Culture

Soil was sampled from the experimental field of Fujian Agriculture and Forestry University in which no rice plants had been grown before. The soil was sterilized at 121°C for 0.5 h, stored at room temperature for 8 h, and then sterilized at 121°C for 0.5 h. This process was repeated three times. The sterilized soil was placed in a sterilized plastic culture box, and each box was filled with 0.8 kg aseptic soil.

*Myxococcus xanthus* and phenolic acid solution, which contained 3, 4-dihydroxybenzoic acid, *p*-hydroxybenzoic acid, vanillic acid, syringic acid, vanillin, ferulic acid, coumarin, benzoic acid, salicylic acid, and cinnamic acid mixed in equal concentrations were added to aseptic soil as treatment groups. Different quantities of phenolic acid mixture (10 μg/kg soil, 20 μg/kg soil, 50 μg/kg soil, and 100 μg/kg soil) were used for the test. Aseptic soil with *M. xanthus* but without any phenolic acid mixture was used as a control. The above treatments were cultured in an artificial climate chamber, and sampling was performed on the 4th and 7th day after treatment initiation. qRT-PCR was used to study the effects of different concentrations of mixed phenolic acids on the population growth of *M. xanthus* added to the soil. Three repeats were set for each test concentration. Each treatment was inoculated with *M. xanthus* (2 mL).

### Differential Expression Analysis of the Chemotaxis-Related Genes of *M. xanthus* in Response to Phenolic Acid

The *M. xanthus* strain stored at −80°C, transferred to 1/2 CTT solid medium for 5 days, and then transferred to 1/2 CTT liquid medium for logarithmic culture. The above bacterial solution was inoculated into 1/2 CTT liquid medium at 1% (v/v), while ferulic acid (0.05 mM and 0.10 mM) and mixed phenolic acids (3, 4-dihydroxybenzoic acid 0.1 μM, *p*-hydroxybenzoic acid 0.4 μM, cinnamic acid 0.2 μM, vanillic acid 0.1 μM, and ferulic acid 0.2 μM) were added to the liquid medium to induce growth. The treatment and control, which contained three repeats, were placed on a shaker and cultured at 250 rpm and 30°C. The bacterial culture samples were collected after 58 h of shaking culture in the ferulic acid treatment group, and 3, 6, 9, 12, 24, and 48 h after treatment initiation in the mixed phenolic acid treatment group. The bacterial total RNA was extracted according to the instructions accompanying Trizol reagent (Takara, China). Subsequently, cDNA was synthesized according to the instructions of the TIANScript RT Kit (Tiangen Biochemical Technology Co., Ltd., Beijing, China). The expression of important differential genes in *M. xanthus* induced by phenolic acid was studied by qRT-PCR with the primers shown in Table 2. The 16S rRNA of myxobacteria was used as the internal standard gene (forward primer sequence: 5′-GACGGTAACTGACGCTGAGAC-3′; reverse primer sequence: 5′-CCCAGGCGGAGAACTTAATGC-3′). Real-time fluorescence analysis was carried out in an Eppendorf realplex4 quantitative PCR instrument. The reaction system was 15 μL, and the configuration followed the operating instructions of RealMasterMix (SYBR Green) (Tiangen Biochemical Technology Co., Ltd., Beijing, China).

**TABLE 2:** Primers of the chemotactic genes of *Myxococcus xanthus* induced by phenolic acid used in qPCR.

Gene	Forward primer 5′ to 3′	Reverse primer 5′ to 3′
*frzA*	GTGAGAACGTGCTCGAAGTG	AAGTTGAGGAGGTTGATGGC
*Frz B*	GTGGACCTCCTCTTCTTC	CAGTGCCTTCCTTGAGT
*frzCD*	CTGCTGGAGGGCTTTGGC	GCGGTCGTCTCGTGGATG
*frzE*	GGCGGCAATCGCTTCGACAA	AACCCGTCCAGCTTGGGCATCT
*frzG*	TTCAGCGGGTGAGTCGTTCG	GCTTCGTCCTGGGCAATGGT
*frzS*	AAAATCCTGATCGTCGAAAG	TTGCCGCAGATGAGGTAG
*csgA*	TGGCAGGTGTTGTTCCTCA	TAGATGCAGTCTGGTCAACCG
*csgB*	AGCCGCAGCAGGTTATGATTT	TGTCACGCGAATAGCCATTT
*csgG*	GAATCTTTCAATGCCGTGACC	TCTTTTGGTTGCCGTCATGT
*fruA*	TGCAACATTAGCAACAGCATTA	TTGGACCTTCTGTTTCACGTT

### Allelopathic Effect of Rhizosphere Special *M. xanthus* on the Targeted Barnyard Grass

#### Evaluation of the Inhibition Effect of *M. xanthus* on Barnyard Grass

##### Seed disinfection

Rice and barnyard grass seeds were separately placed in a tissue culture bottle, sterilized with 70% ethanol for 1 min, and soaked and shaken in ethanol again to achieve full sterilization. The ethanol was then discarded, and the seeds were rinsed with water 3 to 4 times until the smell of ethanol disappeared. Sterilized mercury (1%) was added to the tissue culture bottle, shaken, and seeds were soaked for 15 min. Seeds were rinsed again with sterile water seven to eight times. The sterilized seeds were soaked in sterile water for germination.

##### Inhibitory effect on the grass

We divided this study into three groups with three repeats. The following treatments were established, A: *M. xanthus* with barnyard grass seeds; B: culture medium not inoculated with *M. xanthus* but with barnyard grass seeds; C: *M. xanthus* with rice seeds. The purified strains were coated on plates according to the experimental setting. In the experimental groups, the seeds of allelopathic or non-allelopathic rice accessions and barnyard grass were planted in VY/4 medium, 30 seeds per plate, and were cultured at 28°C for 48 h. Then, the germination rate was calculated.

##### Analysis of the myxobacterial activity to suppress the barnyard grass

Phenolic acids at optimal concentrations (3,4-dihydroxybenzoic acid 0.1 μM, *p*-hydroxybenzoic acid 0.4 μM, cinnamic acid 0.2 μM, vanillic acid 0.1 μM, and ferulic acid 0.2 μM), which were determined in a pre-experiment, were added to the modified 1/2 CTT solid medium, in which the inorganic salt component was reduced to 1/6. The allelochemical group was inoculated with 50 μL of the activated *M*. *xanthus* solution, and its control consisted of only 50 μL sterile water. The group without allelochemicals was also inoculated with 50 μL of the activated *M*. *xanthus* solution. The treatment with only 50 μL water was used as the positive control and that with 50 μL *E. coli* was used as the negative control. Each treatment and control contained three replicates and were incubated at 30°C for 5 days. Under sterilized conditions, the pre-germinated disinfected barnyard grass seeds were evenly sown, 30 seeds per bottle, and the tissue culture bottles were placed in a growth chamber at 30°C for 7 days under 12 h (6:00-18:00) of light and 12 h of dark. Barnyard grass germination and barnyard grass stem length were calculated.

##### Data analysis and Software

The barnyard grass germination and root length were determined on the 7th day after sowing. The inhibitory rate was calculated according to formula (1):

$$\begin{array}{c} \mathrm{Inhibitory}\,\mathrm{rate}\,\left(\mathrm{IR}\right)=\left(1-T/C\right)\times 100\%, \end{array}$$

where C is the measured value of the control group and T is the measured value of the treatment group. IR > 0 indicates inhibition, and IR < 0 indicates a promotion effect. The measured data were statistically analyzed using DPS v 7.05 and the Excel 2003 program.

##### Inhibitory effect of *M. xanthus* culture inoculation on the targeted barnyard grass in soil culture

The sterilized soil was placed in the sterilized plastic culture box; each box was filled with 0.8 kg aseptic soil, then 30 presoaked seeds of the barnyard grass were pre-seeded in the aseptic soil of each box with three replicates. Then, 2 mL, 5 mL, 10 mL, or 20 mL of *M. xanthus* solution and 2 mL of its fermentation broth were added; the broth was the supernatant obtained by centrifuging *M. xanthus* solution for 10 min at 12000 rpm as a treatment. Barnyard grass seeds were planted in aseptic soil without any addition of *M. xanthus* solution and its fermentation broth as a control and were then cultured in an artificial climate box at 37°C for 4 and 7 days. After 4 and 7 days, the germination rates of barnyard grass were calculated, and the effects of different populations of *M. xanthus* on the germination of barnyard grass and rice seeds were determined.

### The Identification and Evaluation of Allelochemicals in the Fermentation Broth With the Induction of Different Phenolic Acids and Their Mixtures Based on GC-QQQMS Analysis

The activation of XAD-16 resin: We weighed the XAD-16 resin (1% w/v) and soak it in double distilled water for 30 min. The resin was collected by filtration and immersed in methanol for 30 min. Then, ultrapure water (50% w/v) was added to soak the resin for further use.

#### Sample Preparation

The activated *M. xanthus* was transferred to 1/2 CTT liquid medium and cultured at 200 rpm, at 30°C for 1 week. Then, the supernatant was collected by centrifugation and passed through a 0.22-μm filter membrane.

The activated XAD-16 resin was added to the supernatant (10%, w/v) and absorbed at 20°C, 200 rpm overnight (12 h), after which the resin was collected by filtration. Methanol was added for extraction at 20°C, 200 rpm overnight, and collected by filtration, followed by extraction with acetone. Then, the methanol extract phase and the acetone extract phase were combined and concentrated by rotary evaporation. The injection bottle for GC-QQQMS was prepared by passing diluted (2 mL) acetone, containing the extract, through a 0.22-μm filter.

The control and other treatments were then measured. The control group included the blank culture medium (CK_0_); PA_0_ refers to the blank culture medium to which the combination of phenolic acids was added. The test group was inoculated with *M. xanthus* on the basis of the corresponding control group: CK_1_ was inoculated with *M. xanthus* in the blank medium; PA_1_ refers to the addition of the combination of phenolic acids to the blank medium followed with *M. xanthus* inoculation.

### Exogenous Addition of Quercetin and Its Effect on Barnyard Grass

#### Determination of the Quercetin Content in the Myxobacterial Fermentation Broth

A quercetin standard (Solarbio Biotechnology Co., Ltd., Beijing, China) was prepared with standard solution concentrations of 3.31 × 10^–4^ mM; 6.62 × 10^–4^ mM; 0.17 × 10^–2^ mM; 0.33 × 10^–2^ mM, and 0.66 × 10^–2^ mM in acetone. The standard solution series and the fermentation liquid sample of *M. xanthus* were injected into a high-performance liquid chromatograph. The chromatograms were recorded, and the peak area was measured. The standard curve was plotted with concentration X (mM) as the abscissa and the chromatographic peak area as Y on the vertical axis.

##### Chromatographic conditions

Column: Shimadzu ODS column (4.6 mm × 150 mm, 5 μm), mobile phase of acetonitrile-water (containing 2% acetic acid), flow rate: 0.8 mL/min, injection volume: 20 μL, column temperature: 25°C, detection wavelength: 360 nm. Eluting procedure: 0 min, acetonitrile 17%, water (containing 2% glacial acetic acid) 83%; 18 min, acetonitrile 40%, water (containing 2% glacial acetic acid) 60%.

#### Effect of Exogenously Added Quercetin on the Growth of Barnyard Grass

According to the content of quercetin detected in the fermentation broth sample determined by HPLC, the linear equation coefficient of determination (R^2^) of the quercetin standard concentration was established: *Y* = 11709X-1118.1 (*R*^2^ = 0.9989). The result indicated that the linear relationship of the quercetin standard concentration was in the range of 3.31 × 10^–4^ mM–0.66 × 10^–2^ mM. The chromatogram of the quercetin standard and PA_1_ sample is shown in Supplementary Figure S1. According to the chromatographic peak area and regression equation, the quercetin content in the sample was 1.06 × 10^–3^ mM.

Macias (1995) proposed that the approximate biological activity range of phenolic acid in higher plants is 10^–6^–10^–5^ M based on the unified biological test standard. Accordingly, a series of quercetin concentrations were set in the 10^–7^–10^–4^ M concentration range, i.e., 1.06 × 10^–4^ mM, 1.06 × 10^–3^ mM, 1.06 × 10^–2^ mM, 0.053 mM, 0.106 mM, and 0.53 mM dosages of quercetin standard were prepared and used for the allelopathic bioassay. The control did not contain quercetin. Each treatment had three replicates and consisted of 30 pre-germinated barnyard grass seeds that were sown in Petri dishes. The tissue culture bottles were placed in a growth chamber at 30°C under light conditions for 12 h (6:00–18:00). After culture for 7 days, the root length and stem length of the barnyard grass were determined, and the inhibitory rate was calculated.

#### Dynamics of Phenolic Acids in Root Exudates of Rice Measured by Solid-Phase Extraction and High-Performance Liquid Chromatography Method

The experiment was conducted in a greenhouse at the Agroecological Institute of Fujian Agriculture and Forestry University in Fuzhou, China. The air temperature ranged from 25°C to 35°C, averaging 30°C during the trials.

Plant materials rice (*Oryza sativa*) accession PI312777 (PI, allelopathic rice) and Lemont (Le, non-allelopathic rice) introduced from the United States were selected as donor plants. Barnyard grass (BYG, *Echinochloa crus-galli* L.) was used as the receiver plant.

Seeds of the two rice accessions and BYG were germinated on seedling trays. Then uniform rice seedlings (2-leaf stage) and BYG seedlings (2-leaf stage) were transplanted into Styrofoam plates with 30 perforated holes (5 × 6 holes with 5 cm × 5 cm row space). The seedlings were stabilized with cotton plugs. The Styrofoam plate was floated on a pot (45 cm × 35 cm × 15 cm) filled with 10 L Hoagland hydroponic solution (served as normal nutrient condition). After 3 days of recovery in the Hoagland hydroponic solution, 30 rice seedlings were transplanted to the Styrofoam plate. In rice/weed mixed culture system, allelopathic rice PI and non-allelopathic rice Le were mixed with BYG at 2:1 ratios with the BYG seedlings in the center surrounded by the rice seedlings in the hydroponic culture system. When rice seedlings reached 3-leaf, 5-leaf, and 7-leaf stages, additional Hoagland nutrient solution was added to each pot to maintain a 10 L solution volume, and the remaining concentration of N, P, and K in hydroponic solutions were detected, then adjusted to the initial concentration levels using NH_4_NO_3_, K_2_SO_4_, and KH_2_PO_4_ (the initial concentration of N, P, and K were 20 mg/L, 5.65 mg/L, and 42.72 mg/L, respectively), furthermore, pH was adjusted to 5.5. A monoculture of BYG under the same condition was used as the control (CK) for each treatment. 250 mL of hydroponic culture was collected for the determination of phenolic acids in rice root exudates. The treatments were conducted in triplicate (He et al., 2012; Li et al., 2017).

##### SPE-HPLC procedure

Reagents and Apparatus: Standards of 3,4-dihydroxybenzoic acid, *p*-hydroxybenzoic acid, vanillic acid, syringic acid, vanilla, ferulic acid, coumarin, benzoic acid, salicylic acid, and cinnamic acid were obtained from the Chinese Institute of Biological Products Control (Beijing, China). Methanol was of HPLC grade from Merck. Ultrapure water from Milli-Q system (Millipore, Bedford, MA, United States) with conductivity of 18.3 MQ was used in all experiments. All solutions were filtered through 0.45 μm membranes (Millipore) and degassed prior to use (Li et al., 2017).

An Agilent 1200 liquid chromatography system, equipped with a quaternary solvent delivery system, an autosampler, and DAD detector, was used. A Zorbax SB-C18 column (150 mm × 4.6 mm, 5 μm) connected with a Zorbax SB-C18 guard column (20 mm × 4 mm, 5 μm) at a temperature of 30°C was applied for all analyses.

##### Solid-phase extraction

Before SPE, hydroponic culture was filtered through a 0.45 μm microporous filter under a weak vacuum. All samples were adjusted pH to 4.0 with 1.0 M HCl, after that, sodium chloride was added to the aqueous solution to obtain an 8% salt solution. The aqueous solution was subjected to SPE using cleanert PEP-SPE cartridges (500 mg; Agela Technologies Inc., Bellefonte, PA, United States) previously activated with ultrapure water (8 mL), methanol (8 mL) and ultra-pure water (4 mL). The phenolic acids were finally eluted with 4 mL of methanol at a rate of 1–2 mL/min. The eluate was dried by nitrogen spraying. The residues were dissolved in 1 mL methanol and a 5 μL aliquot was injected into the liquid chromatography column for HPLC analysis (Li et al., 2017).

##### Chromatographic conditions

Detection wavelength was set at 280 nm. The mobile phase consisted of (A) methanol and (B) 0.1% phosphoric acid (v/v) using a gradient elution of 27% A at 0–6 min, 27–50% A at 6–15 min. Re-equilibration duration was 5 min between individual runs. The flow rate was 1.6 mL/min and aliquots of 5 μL were injected (Li et al., 2017).

#### The Total Concentration of Phenolic Compounds in Root and Leaf Tissues Measured Using the Folin–Ciocalteu Colorimetry Method

Root tissue and leaf tissue were sampled from the recovered transgenic lines and WT plants as described. In a precooled mortar, 1 g of root tissue or leaf tissue frozen with nitrogen liquid was grinded and immersed in 10 mL methanol or sterilized water 10 mL for 24 h at 4°C, and the procedure was repeated twice. These methanolic or water extracts were centrifuged at 13,000 r/min for 15 min, and the supernatants were pooled and stored at 4°C (Shen et al., 2009).

The total content of phenolics was determined using a minimally modified Folin-Ciocalteu method (Singleton et al., 1999). Root tissue or leaf tissue extracts by methanol or water (3.25 ml) were added to test tubes with 0.5 ml of Folin–Ciocalteu’s reagent and 1.25 ml of sodium carbonate (2 M). After mixing, the tubes were allowed to stand for 40 min in dark. Then OD was measured at 760 nm. The total phenolic compounds were expressed in tannic acid equivalents in micromole per liter of root or leaf extracts (Fang et al., 2013; Li et al., 2017).

## Results

### The Population Number of *M. xanthus* in the Rhizosphere Soil of the Allelopathic Rice Accession

**FIGURE 1:**
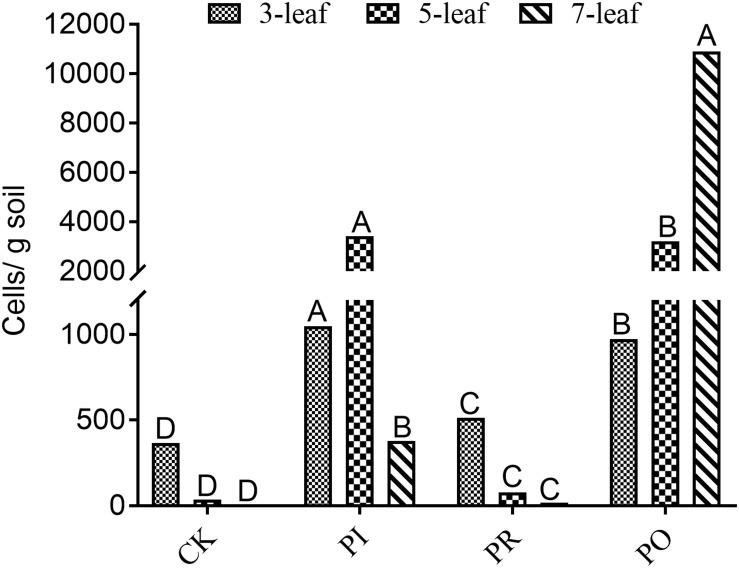
qRT-PCR analysis of soil *Myxococcus* spp. in rhizosphere soil at different leaf stages of the different rice accessions. PI, allelopathic rice PI 312227; PR, *OsPAL2-1-*inhibited allelopathic rice; PO, *OsPAL2-1-*overexpressed allelopathic rice; CK, blank soil. Different letters indicate significant difference (LSD, *p* < 0.05).

As shown in Figure 1, qPCR analysis indicated the same changing trends of the *Myxococcus sp.* population number in the rhizosphere soil of allelopathic rice (PI) and its counterparts (PO, PR) at the 3–7-leaf stages. The population numbers of rhizosphere *Myxococcus sp.* were 1047, 3424, and 378 cells/g soil in the rhizosphere soil of the allelopathic rice accession PI at the 3–7-leaf stages, a significant difference compared with the blank soil (CK). The *OsPAL2-1-*inhibited transgenic line (PR) had 512, 78, and 17 cells/g soil population numbers of *Myxococcus sp.* in rhizosphere soil, significantly lower than the allelopathic rice accession (PI). The reverse was true in the case of the *OsPAL2-1-*overexpressed transgenic line (PO), 974, and 3209 cells/g soil *Myxococcus sp.* population numbers in rhizosphere soil at the 3-and 5-leaf stages, had no significant difference compared with the of PI312777, and contained the largest population number of *Myxococcus sp.*, 10899 cells/g soil, in rhizosphere soil at the 7-leaf stage, which resulted in the highest population numbers of *M. xanthus* in rhizosphere soil at the 3–7-leaf stages.

### *Myxococcus xanthus* Isolated and Identified From the Rhizosphere Soil of Allelopathic Rice

The biological characteristics of the strains were as follows: the fruiting body induced from rabbit feces was yellow and spherical, and the secondary colony obtained by line culture was milky white, raised in the middle with an irregular edge (Supplementary Figure S2A). In the early stage, the colony showed a transparent or translucent milky white irregular shape, and the edge was irregular. After about 48 h of culture, the fruiting body began to appear in the colony; the fruiting body was yellow and granular, and the colony also changed to yellow. In addition, the viscosity increased, becoming transparent or translucent, and the edge of the colony was irregular (Supplementary Figures S2B,C). The 16s rRNA fragment that amplified from the genomic DNA of the above strains was purified and sequenced. Based on Blast sequence alignment combined with its colony morphology, this strain was identified as *M. xanthus* with the highest similarity.

### *In vitro* Interactions of *M. xanthus* With Phenolic Acid Allelochemicals in Rice

**FIGURE 2:**
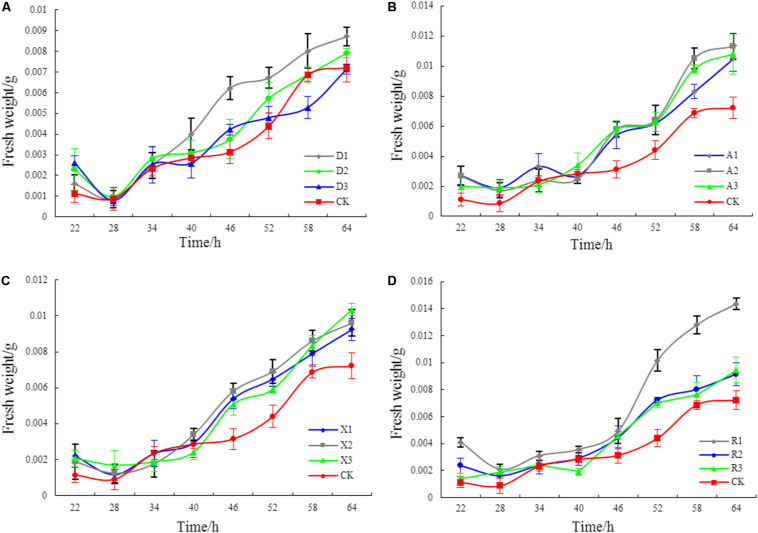
The effects of different phenolic acids on the growth of myxobacteria. **(A)** Effects of cinnamic acid at different concentrations on myxobacteria (R1: 0.12 mM; R2: 0.20 mM; R3: 0.28 mM). **(B)** Effects of ferulic acid at different concentrations on myxobacteria (A1: 0.05 mM; A2: 0.1 mM; A3: 0.15 mM). **(C)** Effects of hydroxybenzoic acid at different concentrations on myxobacteria (D1: 0.40 mM; D2: 0.50 mM; D3: 0.60 mM). **(D)** Effects of vanillic acid at different concentrations on myxobacteria (X1: 0.02 mM; X2: 0.10 mM; X3: 0.18 mM).

**FIGURE 3:**
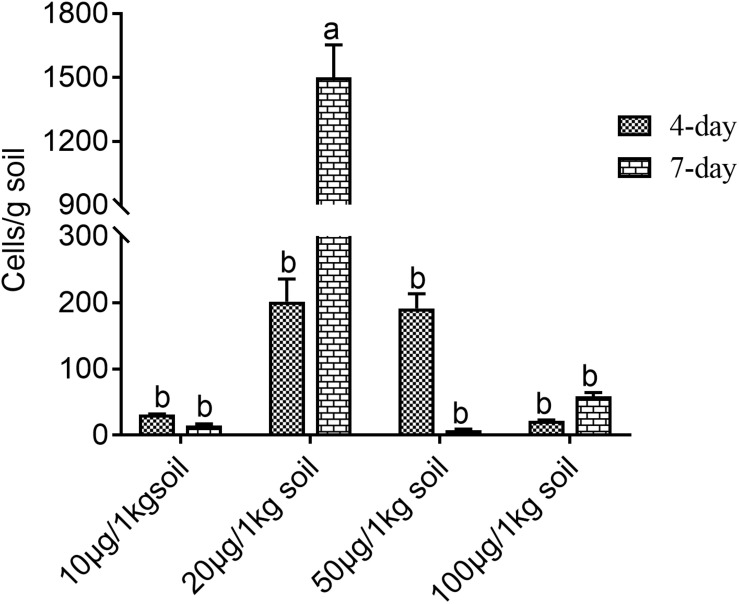
Effects of mixed phenolic acids at different concentrations on the population growth of *Myxococcus xanthus* under sterile soil culture conditions. Different letters indicate significant difference (LSD, *p* < 0.05).

Figure 2 shows that different phenolic acid allelochemicals showed various effects on the growth of the myxobacteria, indicating that cinnamic acid at 0.12 mM and ferulic acid at 0.05−0.1 mM significantly promoted the proliferation of the myxobacteria. However, no significant difference was found for the effect of hydroxybenzoic acid and vanillic acid on the bacteria at 0.4 mM and at 0.02–0.18 mM, respectively, in the *in vitro* interaction test. The influence of the phenolic acid mixture at different concentrations on the population growth of *M. xanthus* showed a tendency of an increasing and then a decreasing pattern under soil culture conditions (Figure 3). There was no significant difference in the population of *M. xanthus* in the coculture with different concentrations of phenolic acid mixture for 4 days. However, after 7 days of coculture, the population of *M. xanthus* was significantly different and was the largest in the soil with the 20 μg/kg of phenolic acid mixture. These findings suggested that the population growth of myxobacteria was closely related to the concentration of the mixed phenolic acids in soil. However, there was an optimal concentration range in terms of the action effect, i.e., too low or too high a concentration of the phenolic acid mixture, added by external sources or secreted by the root system of rice accessions, was unfavorable for the growth of the special microbial flora in the rice rhizosphere.

### Differential Expression Analysis of the Related Chemotaxis Genes of *M. xanthus* in Response to Phenolic Acids

**FIGURE 4:**
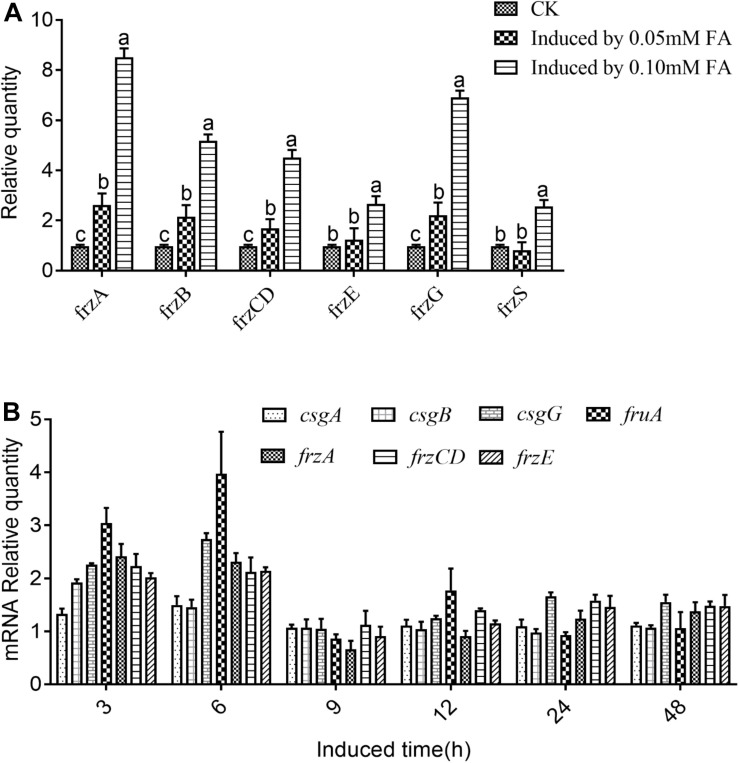
Relative expression quantity of the genes encoding frz multigene family members in *Myxococcus xanthus* under ferulic acid treatment **(A)** and the relative expression quantity of chemotaxis-related genes in *M. xanthus* induced by mixed phenolic acids **(B)**. Significance refers to a comparison of the expression level of the gene under different phenolic acid concentrations compared with the control. Those significances in the figure marked with the same letter in the same column indicate no significant difference at the 5% level (LSD method, *P* < 0.05).

Further analysis demonstrated that a single phenolic acid or mixture of phenolic acids could effectively induce the up-regulated expression of the related chemotaxis genes in *M. xanthus* at different levels (Figures 4A,B). Gene expression changes in each member of the *Frz* family were detected in *M. xanthus* cocultured with different concentrations of ferulic acid for 58 h, as shown in Figure 4A. The result showed that the changes in *FrzS* gene expression were non-significant between the coculture treatment with 0.05 mM ferulic acid and the control without the addition of any phenolic acids. However, the up-regulated rate of *FrzS* in *M. xanthus* in the cocultured treatment with 0.10 mM ferulic acid was 2.56-fold higher than that of the control. Furthermore, the same situation was observed for gene expression levels of four other *Frz* family members, showing that *FrzA, FrzB, FrzCD*, and *FrzG* in *M. xanthus* were significantly higher under the coculture with ferulic acid at 0.05 mM compared with the control, indicating that the up-regulated rate was 2.60-fold, 2.16-fold, 1.75-fold, and 2.23-fold higher. However, the change in the *FrzE* gene was not significant between the coculture treatment and the control. The up-regulated expressions of the *FrzA, FrzB, FrzCD, FrzE*, and *FrzG* genes in *M. xanthus* under the cocultured treatment with 0.10 mM ferulic acid were also increased by 8.53-fold, 5.16-fold, 4.52-fold, 2.70-fold, and 6.91-fold, respectively, compared with the control without coculture with 0.10 mM ferulic acid (Figure 4A).

qPCR analysis of the related chemotactic genes was conducted on *M. xanthus* cocultured with the mixtures of phenolic acids, as shown in Figure 4B. It was found that the up-regulated rate of each gene was higher than that of the control, and the highest gene expression level was detected in *M. xanthus* cocultured with phenolic acid for 3–6 h. *CsgA* was up-regulated in *M. xanthus* cocultured with the phenolic acid, by 2.09-fold and 2.76-fold compared with the control at 3 h and 6 h, respectively, and then the expression level dropped. *FruA* was also up-regulated in the coculture treatment, by 3.17-fold and 3.19-fold, compared with the control at 3 h and 6 h, respectively, and then almost returned to its normal expression level. The expression level of *FrzCD* in *M. xanthus* cocultured with the phenolic acid at 3 h and 6 h was also increased, by 1.95-fold and 1.34-fold, respectively, compared with the control. However, in the coculture treatment for 9 h, the *FrzCD* expression level in *M. xanthus* decreased and was then up-regulated 1.99-fold compared with the control at 48 h. *CsgB*, *CsgG*, *FrzA*, and *FrzE* in *M. xanthus* showed a similar expression trend in response to the coculture treatment, indicating the highest up-regulated rate of those genes in *M. xanthus* cocultured with the phenolic acid at 3 h was 2.99-fold, 5.61-fold, 2.48-fold, and 3.48-fold, respectively, compared with the control without the addition of the phenolic acid. Subsequently, the expression levels of these genes were decreased gradually in the coculture treatment as shown in Figure 4B.

### Allelopathic Effect of Rhizosphere Special *M. xanthus* on the Targeted Barnyard Grass

**FIGURE 5:**
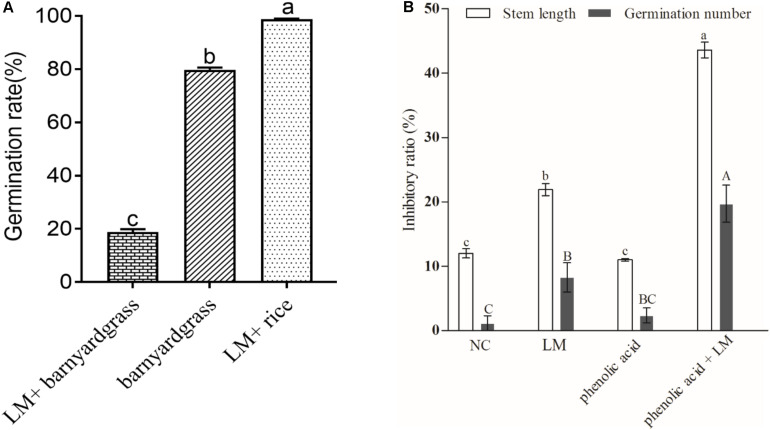
The average germination rate of barnyard grass and rice seeds under different coculture conditions **(A)** and the inhibitory effect of myxobacteria induced by the phenolic acid mixture on barnyard grass growth **(B)**. **(A)** LM + barnyard grass refers to the target barnyard grass seeds cocultured with the screened myxobacterial strain in the medium; barnyard grass refers to barnyard grass seeds incubated in the medium; LM + rice refers to allelopathic rice seeds cocultured with the screened myxobacterial strain in the medium. **(B)** NC, negative control; LM, coated with myxobacteria only in the medium; Phenolic acid coated only with the phenolic acid mixture in the medium; Phenolic acid + LM, Myxobacteria in the medium with the mixture of phenolic acids. Different letters indicate significant differences (LSD, *p* < 0.05).

The result of the bioassay showed that for the targeted barnyard grass seeds, which were cocultured with the screened myxobacterial strain in the medium, the germination rate was 18.89%, i.e., a significant decrease of 61.08% compared with the control, as shown in Figure 5A. We also observed a significant allelopathic interaction between rhizosphere special myxobacteria and phenolic acids in coculture with the targeted barnyard grass (Figure 5B). The inhibition rate of barnyard grass seed germination and the stem length of the target plant was 8.25 and 21.89%, respectively, in the plate (LM) containing *M. xanthus*. The inhibition rate of barnyard grass seed germination and the target stem length increased to 19.70 and 43.59%, respectively, on the plate (LM + phenolic acid) containing *M. xanthus* and the phenolic acid mixture; these values were much higher than those (2.34%, 10.98%) for the plate containing only the phenolic acid mixture. *Escherichia coli* was used as control bacteria in this study (negative control, NC). The results showed that barnyard grass germination on the plate containing only *E. coli* was not significantly different from that of the control, and the target stem length was lower than that of the control but significantly higher than that in the other treatments (Figure 5B). The findings suggested that the combination of the phenolic acid mixture with the myxobacterial strain had the most significant inhibitory effect on the germination and seedling growth of the targeted barnyard grass, showing that the interaction between phenolic acid allelochemicals and *M. xanthus* had a significant impact on the allelopathic potential of rice.

To investigate the allelopathic inhibitory effect of *M. xanthus* on barnyard grass in soil, the effects of the treatments with *M. xanthus* and its fermentation broth at different added amounts on barnyard grass seed germination were determined under sterile soil conditions. The result showed that the inhibitory effect of added inoculant (5 mL) on the germination of barnyard grass seeds was significantly higher than in any other treatments, indicating a 53.4% inhibition rate in the suppression of the target weed at 4 and 7 days. It was also found that when we added 2 mL of the fermentation broth to the soil, the inhibition rate significantly reached 78.80 and 75.80% at 4 and 7 days, respectively, i.e., significantly higher than that in the addition treatment with 5 mL of the myxobacterial inoculant solution added to the sterile soil pre-seeded with the target barnyard grass (Table 3). These findings suggested that the effect of the myxobacteria on the growth of barnyard grass was based on the population density of the target plants in the soil, and the fermentation broth of *M. xanthus* showed the highest allelopathic potential for suppression of the target weeds, but this needs further study.

**TABLE 3:** Effects of rhizosphere special myxobacteria and its fermentation broth at different dosages on the germination of barnyard grass added to sterile soil.

Co-culture days	Treatment	Germination rate (%)	Standard deviation	Inhibition rate (%)	Standard deviation
	CK	97.80	0.1361 A	2.30	0.1361 C
4D	2 mL M	71.00	0.0346 AB	29.00	0.0346 BC
	5 mL M	46.60	0.135 BC	53.40	0.135 AB
	10 mL M	51.20	0.1015 BC	48.80	0.1015 AB
	20 mL M	60.00	0.2000 ABC	40.00	0.2000 BC
	2 mL F	22.20	0.1015 C	78.80	0.1015 A
7D	CK	100.00	0.1155 A	0.00	0.1155 C
	2 mL M	73.40	0.0651 AB	26.70	0.0651 BC
	5 mL M	46.60	0.135 BC	53.40	0.1350 AB
	10 mL M	51.20	0.1015 BC	48.80	0.1015 AB
	20 mL M	73.40	0.2000B	26.60	0.2000 BC
	2 mL F	24.20	0.0751 C	75.80	0.0751 A

*CK, blank barnyard grass. M refers to the myxobacterial (*M. xanthus*) solution with barnyard grass. F refers to the fermentation broth of *M. xanthus* after 3 days, at a 2-mL dosage with barnyard grass. Different capital letters A, B, and C indicate significant differences (LSD, *p* < 0.01).*

### The Mechanism of Suppression of Barnyard Grass Under Coculture Treatment With Myxobacteria and the Optimal Mixture of Different Phenolic Acids

The analyses and identification of allelochemicals in different coculture samples were conducted by GC-QQQMS, and the results showed that the quantity and composition of the secondary metabolites in the myxobacterial fermentation broth were quite different under different conditions, such as in the medium with the mixture of the phenolic acids (PA_0_) or with *M. xanthus* and the optimal mixture of phenolic acid (PA_1_). Numerous compounds, such as ketones, esters, and aromatic and sulfur compounds, significantly increased in the coculture sample from the treatment of *M. xanthu*s with the mixture of the different phenolic acids (PA_1_) compared with the sample treated only with the phenolic acid (PA_0_), for the determination and comparison of allelopathic inhibitory effects on the target weeds. Compared with those in the CK_0_ and CK_1_ treatments, the ketones and esters increased and alkanes/alkenes increased significantly under the treatment of PA1, which suggested that the optimal mixture of different phenolic acids was able to promote the production of secondary metabolites in the specific myxobacteria under the coculture condition (Supplementary Tables S1–S5), which increased its allelopathic potential of the suppression of the target weeds.

Further analysis showed that quercetin, a well-known allelochemical, was detected in the sample under PA_1_ treatment, but not in the other treatment (PA_0_), suggesting that it might interact with other toxic ketones and quinones to influence the herbicidal potential of the myxobacteria, as shown in Table 4.

**TABLE 4:** Identification of the secondary metabolites in the fermentation broth of the rhizosphere special *Myxobacteria* in the treatment with the phenolic acid (PA_1_).

Treatments	Molecular Formula	Name
PA_1_	C_10_H_16_O	2 – decalin ketone
	C_4_H_2_BrN_5_O	5- brompyrazole - [3,4-d] -s- triazine -4(3H)- ketone
	C_16_H_24_O	Allyl ionone
	C_13_H_12_O_5_S	2-(2, 5-dioxo tetrahydrofuran-3-group) thio-3,5, 6-trimethyl-p-quinone
	C_19_H_18_O_6_	6, 7-dimethoxy -3- [2-(2-methoxy phenyl)- 2-oxyethyl] -1(3H) – benzofuranone
	C_30_H_52_O	cycloxylenol
	C_15_H_12_O_2_	1-phenyl-3-m-hydroxyphenyl-allenone
	C_16_H_13_NO_4_	*Trans* -4′- methoxy -4- nitrochalcone
	C_22_H_21_N_3_O	2, 3-dihydro-3 -(4-dimethylaminophenyl)- 2-phenyl-quinazoline -4(1H)- ketone
	C_18_H_22_N_2_O	4- [[4-(diethyl amino)-2, 6-dimethyl-phenyl] imino] -2, 5-cyclohexadiene -1- ketone
	C_5_H_4_C_*l*__2_O_3_	3, 4-dichloro-5-methoxy -2(5H) – furanone
	C_15_H_10_O_7_	**Quercetin**
	C_14_H_22_O_2_	4-(3-hydroxy-2,6, 6-trimethyl-cyclohexan-1-alkene) pente-3-alkene-2-ketone

**FIGURE 6:**
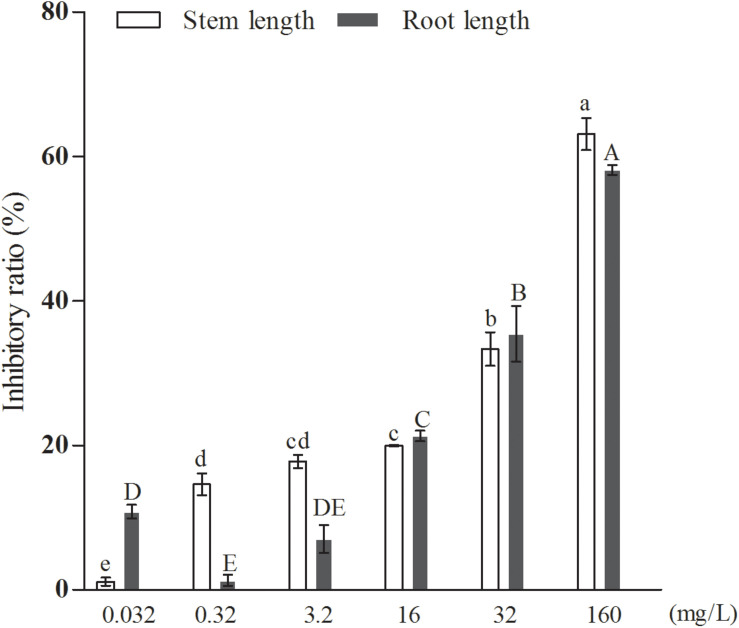
The effects of exogenously added quercetin on the growth of barnyard grass. Different letters indicate significant differences (LSD, *p* < 0.05).

Further results, shown in Figure 6, indicated that the inhibitory effect of the added quercetin at a concentration of 1.06 × 10^–3^ mM on barnyard grass stem length and root length was 12.68 and 14.17%, respectively, and all of them were significantly different compared with the control. When the concentration of the added quercetin was reduced 10 times, i.e., 1.06 × 10^–4^ mM, it had no inhibitory effect on barnyard grass stem length, while the inhibitory effect on the root length of the target plant was significant, with an inhibition rate of 32.91% in comparison with the control. When the dosage was increased by 10, 50, 100, and 500 times, i.e., in the coculture with the added quercetin at 1.06 × 10^–2^ mM, 0.053 mM, 0.106 mM, and 0.53 mM, respectively, the inhibition rate increased with the increase of the treated concentrations of quercetin in the suppression of the target weeds, indicating that the inhibition rates were 16.59, 17.80, 29.82, and 47.06% with respect to the suppression of the stem length of the target plant and 30.55, 34.59, 36.30, and 60.59% for the root length of the target plan. Based on these findings, it can be concluded that the effect of quercetin on the root length of the target plant was greater than that on the stem length of the target plant (barnyard grass).

## Discussion

Allelochemicals from root exudates have a significant influence on the biodiversity of soil microorganisms, and specific microbes interact with allelochemicals increasing the allelopathic inhibition on target weeds (Lin et al., 2007, 2011; Kong et al., 2008; Qu and Wang, 2008; Shi et al., 2011). The allelopathic rice accession PI attracted higher numbers of microbial populations in the rhizosphere soil than that of non-allelopathic rice accession Le, of which seven were identified as myxobacteria (Xiong et al., 2012). The higher contents of phenolic acids in the PI than in the Le was determinate in this study (Supplementary Table S6) and these compounds played roles in promoting the proliferation of specific microbe, i.e., *M. xanthus*. The indoor experiment showed that the exogenous ferulic acid (FA) increased the population of *M. xanthus*, and the combined activity of FA and *M. xanthus* exhibited the highest allelopathic inhibition of the barnyard grass, which was higher than that under the individual application of FA or *M. xanthus* (Fang et al., 2015).

Phenolic acid is mainly synthesized in the phenylalanine metabolism pathway (Ferrer et al., 2008), and this is one of the dominant pathways for the synthesis of rice allelochemicals; *OsPAL* is the first key catalyticase in the pathway. The activity of *OsPAL* contributes to the intensity of metabolism. Overexpression of *OsPAL2-1* in PI resulted in an enhanced rhizosphere microbial population and allelopathic inhibition of barnyard grass, whereas silencing of *OsPAL2-1* in PI led to a decrease of allelopathic inhibition and microbial population and diversity (Fang et al., 2015; 2013), indicating the vital role of *OsPAL2-1* in the regulation of rice allelopathic potential. *OsPAL2-1* of allelopathic rice was reported to be highly sensitive to environmental stress (such as low nitrogen and high weed density) compared with non-allelopathic rice (Wang et al., 2010; Lin, 2013; Zhang et al., 2018). An increase in the *OsPAL2-1* expression level resulted in a significant increase in the secretion of secondary metabolites, especially phenolic acids (Supplementary Table S7), which contributed to increased microbial populations in the rhizosphere.

Our comparative study on the difference in the rhizosphere microbial population of PI, PR, and PO, shows that *Myxococcus* spp. was significantly decreased in the rhizosphere soil of the *OsPAL2-1* gene-silenced rice line PR compared with its wild-type of PI (Figure 1). The *OsPAL2-1*-overexpressed transgenic line (PO) had significantly greater abundance of *M. xanthus* compared with PI (Figure 1), which was related to the process involved in the influence of the *OsPAL2-1* gene expression abundance on the synthesis and secretion of allelopathic rice rhizospheric phenolic acids, leading to proliferation of the myxobacteria in rhizospheric soil. The results *in vitro* interaction and sterile soil tests confirmed that the proliferation of *M. xanthus* could be promoted by some phenolic acids such as ferulic acid or a phenolic acid mixture in a given range of active dosages. Previous studies reported that myxococcales are known as metabolic factories and can produce a large number of secondary metabolites and can inhibit the germination of weeds and the proliferation of various harmful bacteria and fungi (Weissman and Müller, 2009). Ye et al. (2020) also documented that myxobacterium *Corallococcus* sp. strain EGB modified the soil microbial community structure and reduced the quantities of *Fusarium oxysporum* f. sp. *cucumerinum* in the soil by predation, which makes the beneficial microbial community stable and reduces the occurrence of diseases.

Myxobacterium occur in a complex soil environment and rely on signaling pathways to regulate their cellular behavior to adapt to changing environments (such as physical, chemical, and biological stimuli). This behavior is called quorum sensing (QS). Some of the bacteria produce chemical signaling substances, whereas other cells use the chemical signals to determine population density and changes in the surrounding environment and respond accordingly by multiplying or by producing toxins. However, myxobacteria release signals that are not acyl-homoserine lactones (AHLs) or autoinducer-2; however, they produce and use the A-signal and C-signal (Whitworth, 2008) in response to environmental stress. Mcvittie first identified intercellular signals during myxobacterial growth using different mutants in 1962 (Mcvittie et al., 1962). So far, at least five kinds of intercellular signals have been identified: A (Asg), B (Bsg), C (Csg), D (Dsg), and E (Esg) (Downard et al., 1993; Kaiser and Warrick, 2011). The chemical properties of the A-signal and C-signal have been determined; both play important roles in the aggregation of vegetative cells and the early development of fruiting bodies.

In the present study, the chemotactic motility of the rhizosphere special myxobacterial strain could be directly observed under the induction of the phenolic acids using the slide method (data not shown). Further analysis demonstrated whether a single phenolic acid or mixture of phenolic acids could effectively induce the unregulated expression of chemotaxis-related genes in *M. xanthus* (Figures 4A,B). The results showed that ferulic acid at an appropriate concentration effectively stimulated the *Frz* family member automatic chemotaxis system of *M. xanthus*, which is conducive to the completion of the chemotactic behavior of bacteria, thus promoting the aggregation of the specific bacteria in the rhizosphere soil of the allelopathic rice accession. The findings suggest that the regulation of the *OsPAL2-1* gene could significantly mediate the interaction of allelopathic rice with *M. xanthus* in rhizosphere soil, together with increased phenolic acid exudation in this process, which might play an important role in the chemotactic aggregation of the special microbe in the rhizosphere soil of allelopathic rice. In cucumber, the myxobacterium *Corallococcus* sp. was found migrating toward the roots and root exudates of the cucumber plants via chemotaxis (Ye et al., 2020). In this study, the phenolic acid mixture secreted from the allelopathic rice could promote the production of secondary metabolites of the myxobacteria (Supplementary Table S1), of which quercetin was determined as the main allelochemical, suggesting that it might interact with other toxic ketones and quinones to influence the herbicidal potential of the myxobacteria (Table 4).

## Conclusion

In conclusion, this study suggested that allelopathic rice could affect the metabolism of phenolic acid allelochemicals via the regulation of the key gene *OsPAL2-1*. The phenolic acids secreted by allelopathic rice into soil could mediate the myxobacteria to gather themselves together in the rhizosphere, through which the production of a large number of secondary metabolites with allelopathic activity begins. One such example is quercetin, which is a potential allelochemical, generating from the FA-induced *M*. *xanthus* cultured medium and playing role in suppression of weed germination and growth. This could be considered an underlying reaction of rhizosphere biochemistry in rice allelopathy. However, the manner in which myxobacteria regulate their own physiological and biochemical reactions to achieve a high aggregation effect (quorum sensing effect) in a short time remains unknown. Therefore, in-depth answers to these questions are of great theoretical and practical significance for the ultimate understanding of the rhizosphere biological process and molecular ecological mechanism of rice allelopathy, which can also provide a scientific basis for the research and development of safer and more efficient regulation methods to improve the allelopathic inhibition of weeds in paddy fields.

## Data Availability Statement

All datasets generated for this study are included in the article/Supplementary Material.

## Author Contributions

WL and YiL conceived the study and wrote the manuscript. YiL, XJ, YuL, and XZ performed the experiments. YiL and LX performed the statistical analyses. MK revised the manuscript. All of the authors discussed the results and commented on the manuscript, and they have approved it for publication.

## Conflict of Interest

The authors declare that the research was conducted in the absence of any commercial or financial relationships that could be construed as a potential conflict of interest.

## Supplementary Material

FIGURE S1 HPLC of quercetin and the sample.

FIGURE S2 The morphology of fruiting bodies **(A)** of the strain induced by rabbit feces and the morphology of fruiting bodies **(B)** and the colony **(C)** of the purified strain.

TABLE S1 The secondary metabolites of *Myxobacteria* induced by different allelochemicals.

TABLE S2 The secondary metabolites in the blank culture medium (CK_0_).

TABLE S3 The secondary metabolites of *M. xanthus* in the blank culture medium (CK_1_).

TABLE S4 The secondary metabolites in the blank culture medium with the mixture of phenolic acids (PA0).

TABLE S5 The secondary metabolites of *M. xanthusin* the coculture with different phenolic acid mixtures (PA_1_).

TABLE S6 Concentrations of phenolic acids in root washings of Le and PI in a normal hydroponic culture system and under BYG stress (μg/L).

TABLE S7 Concentration of total phenolics in root and leaf tissues measured using the Folin–Ciocalteu method (mg/g Root/Leaf fresh weight).
