# Supplementary material for: OsPAL2-1 Mediates Allelopathic Interactions Between Rice and Specific Microorganisms in the Rhizosphere Ecosystem
Source: Front Microbiol. 2020 Jul 23;11:1411. doi: 10.3389/fmicb.2020.01411 (PMC7391800; doi:10.3389/fmicb.2020.01411)
Supplement: FIGURE S1 — HPLC of quercetin and the sample. [file Table_1.DOCX]

***OsPAL2-1* mediates allelopathic interaction between rice and specific microorganisms in rhizosphere ecosystem**

**Running title: *OsPAL2-1* regulates rice allelopathic potential**

**Yingzhe Li ^1, 3^, Xin Jian ^1, 3^, Yue Li ^1, 3^, Xiaomei Zeng ^1, 3^, Lining Xu ^1, 3^, Muhammad Umar Khan ^1, 3^, Wenxiong Lin ^1, 2, 3^ ^*^**

^1^ Fujian Provincial Key Laboratory of Agroecological Processing and Safety Monitoring, College of Life Sciences, Fujian Agriculture and Forestry University, Fuzhou 350002, P. R. China.

^2^ Key Laboratory of Ministry of Education for Genetics, Breeding and Multiple Utilization of Crops, College of Agriculture, Fujian Agriculture and Forestry University, Fuzhou 350002, P. R. China.

^3^ Key Laboratory of Crop Ecology and Molecular Physiology (Fujian Agriculture and Forestry University), Fujian Province University, Fuzhou 350002, P. R. China.

**Table**

**Table S1** The secondary metabolites of *Myxobacteria* induced by different allelochemicals

| Allelochemicals | CK_0_ | CK_1_ | PA_0_ | PA_1_ |
| --- | --- | --- | --- | --- |
| Alkane/Alkene | 4 | 24 | 4 | 7 |
| Alcohols | 1 | 4 | 4 | 5 |
| Aldehyde | 1 | 1 | 1 | 0 |
| Ketones | 3 | 6 | 6 | 10 |
| Esters | 14 | 20 | 12 | 21 |
| Aromatics | 4 | 3 | 2 | 6 |
| Sulfur compounds | 1 | 2 | 3 | 5 |
| Others | 16 | 21 | 20 | 17 |
| Totality | 44 | 81 | 44 | 71 |

Note: CK0: Blank medium; CK1: *M. xanthus*; PA0: Phenolic acids mixture; PA1: Phenolic acid mixed with *M. xanthus*.

**Table S2** The secondary metabolites in the blank culture medium (CK_0_)

| CAS Number | molecular formula | Structural Formula | RT (min) |
| --- | --- | --- | --- |
| 2065-00-1 | C_8_H_10_O_3_ | 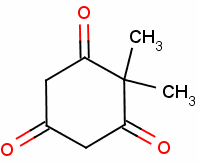 | 19.735 |
| 84-69-5 | C_16_H_20_O_4_ | 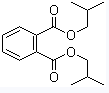 | 20.335 |
| 2612-14-8 | C_63_H_88_N_12_O_16_ | 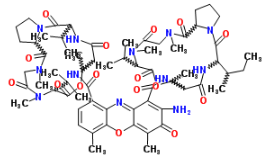 | 20.625 |
| 84-74-2 | C_16_H_22_O_4_ | 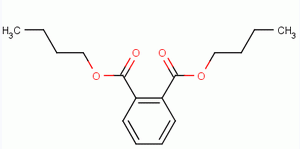 | 22.215 |
| 63331-02-2 | C_9_H_19_NO_3_SSi | 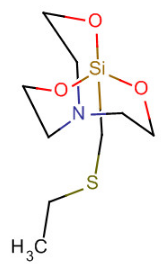 | 25.260 |
| 19102-90-0 | C_18_H_34_O_4_ | 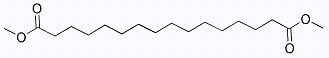 | 27.515 |
| 2097-16-7 | C_8_H_17_NO_3_Si | 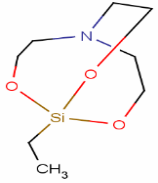 | 28.72 |
| 119-47-1 | C_20_H_12_ | 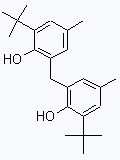 | 30.065 |
| 511-12-6 | C_33_H_37_N_5_O_5_ | 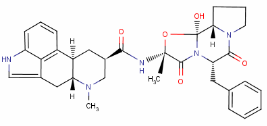 | 30.16 |
| 117-81-7 | C_24_H_38_O_4_ | 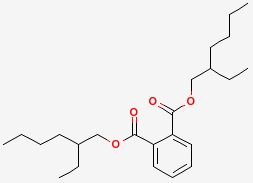 | 31.905 |
| 301-02-0 | C_18_H_35_NO | 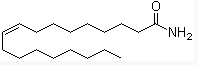 | 35.58 |
| 79-47-0 | C_3_ClF_5_ | 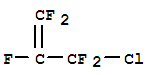 | 40.835 |
| 13159-28-9 | C_30_H_48_O_2_ | 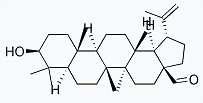 | 42.530 |
| 60-79-7 | C_1_9H_23_N_3_O_2_ | 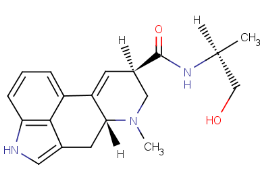 | 42.830 |
| 14633-54-6 | C_9_H_10_S | 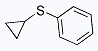 | 46.830 |
| 40515-73-9 | C_15_H_21_NO_5_ | 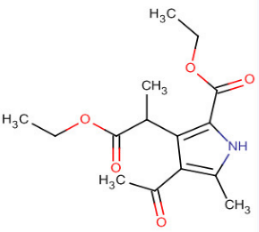 | 48.540 |

**Table S3** The secondary metabolites of *M. xanthus* in the blank culture medium (CK_1_)

| CAS Number | molecular formula | Structural Formula | RT (min) |
| --- | --- | --- | --- |
| 485-31-4 | C_15_H_18_N_2_O_6_ | 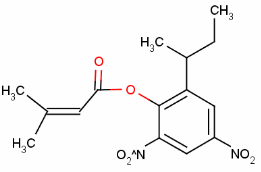 | 5.005 |
| 55299-24-6 | C_18_H_19_ClN_2_OSi | 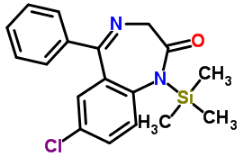 | 7.210 |
| 84-69-5 | C_16_H_20_O_4_ | 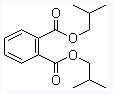 | 20.320 |
| 82304-66-3 | C_17_H_24_O_3_ | 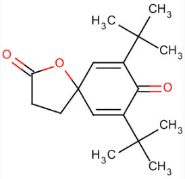 | 21.270 |
| 884-36-6 | C_13_H_24_O_2_ | 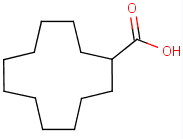 | 23.485 |
| 60-33-3 | C_18_H_32_O_2_ | 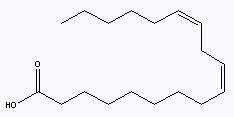 | 24.615 |
| 469-61-4 | C_15_H_24_ | 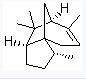 | 26.030 |
| 301-02-0 | C_18_H_35_NO | 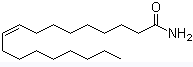 | 29.540 |
| 78987-68-5 | C_11_H_11_C_l3_O_2_ | 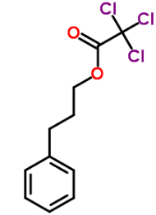 | 30.150 |
| 86702-46-7 | C_15_H_23_NO_3_ | 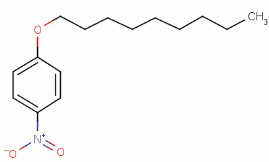 | 35.245 |
| 77573-30-9 | C_17_H_24_O_5_ | 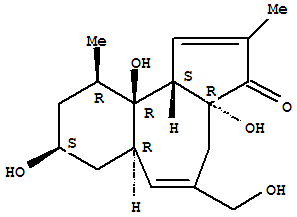 | 40.655 |
| 1022-86-2 | C_15_H_11_NO | 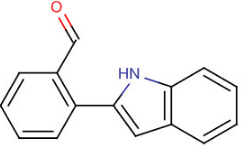 | 43.655 |
| 1757-18-2 | C_12_H_14_C_l3_O_3_PS | 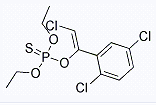 | 43.730 |
| 26264-02-8 | C_25_H_44_O_6_ | 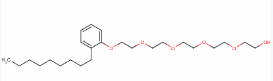 | 43.830 |
| 7235-40-7 | C_40_H_56_ | 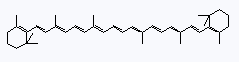 | 46.345 |
| 60705-62-6 | C_44_H_56_O_4_ | 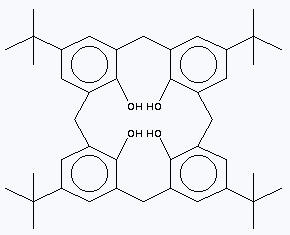 | 46.675 |

**Table S4** The secondary metabolites in the blank culture medium with the mixture of phenolic acids (PA_0_)

| CAS Number | molecular formula | Structural Formula | RT (min) |
| --- | --- | --- | --- |
| 84-69-5 | C_16_H_20_O_4_ | 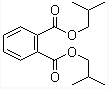 | 20.335 |
| 2065-00-1 | C_8_H_10_O_3_ | 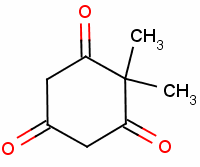 | 22.355 |
| 73579-08-5 | C_7_H_18_N_2_ | 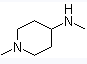 | 22.390 |
| 2097-16-7 | C_8_H_17_NO_3_Si | 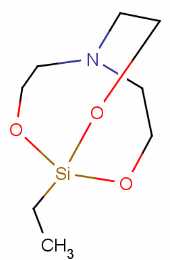 | 25.260 |
| 35479-44-8 | C_20_H_30_O_2_Si_2_ | 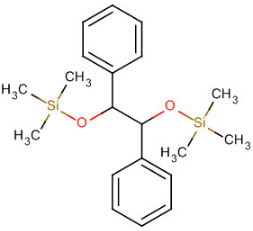 | 35.230 |
| 3618-19-7 | C_16_H_30_O_4_Si_3_ | 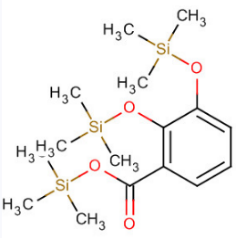 | 36.375 |
| 588-68-1 | C_14_H_12_N_2_ | 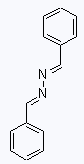 | 40.805 |
| 1023-91-2 | C_16_H_16_O_2_ | 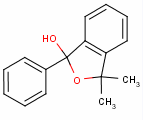 | 42.520 |
| 6777-09-9 | C_12_H_16_O_3_ | 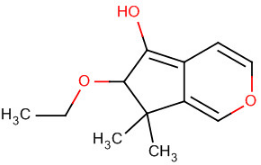 | 43.750 |
| 2784-73-8 | C_19_H_21_NO_4_ | 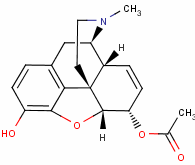 | 44.140 |
| 108154-54-7 | C_18_H_11_ClO_4_ | 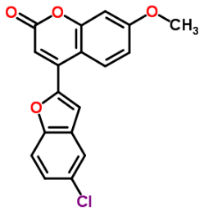 | 44.195 |
| 3618-20-0 | C_16_H_30_O_4_Si_3_ | 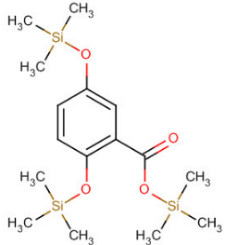 | 45.645 |
| 5508-47-4 | C_23_H_28_ClN_3_O_3_ | 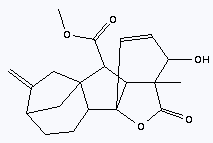 | 48.030 |
| 38472-90-1 | C_12_H_14_O_4_ | 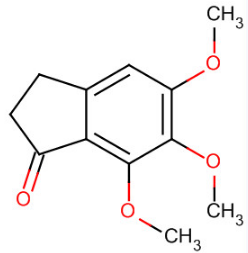 | 48.090 |
| 99-54-7 | C_6_H_3_C_l2_NO_2_ | 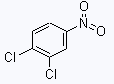 | 48.200 |

**Table S5** The secondary metabolites of *M. xanthus* in the coculture with different phenolic acid mixtures (PA_1_)

| CAS Number | molecular formula | Structural Formula | RT (min) |
| --- | --- | --- | --- |
| 3682-17-5 | C_9_H_9_NO_3_ | 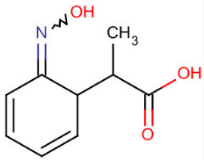 | 3.910 |
| 4832-17-1 | C_10_H_16_O | 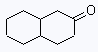 | 26.755 |
| 552-02-3 | C_15_H_26_O | 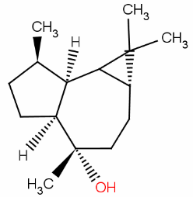 | 35.745 |
| 98258-72-1 | C_17_H_14_O_3_ | 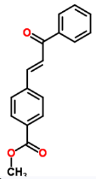 | 37.285 |
| 126848-01-9 | C_13_H_12_O_5_S | 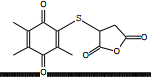 | 38.470 |
| 2315-62-0 | C_20_H_34_O_4_ | 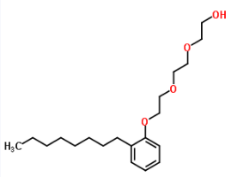 | 41.295 |
| 22316-93-4 | C_16_H_15_N_3_O | 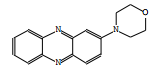 | 42.515 |
| 3618-20-0 | C_16_H_30_O_4_Si_3_ | 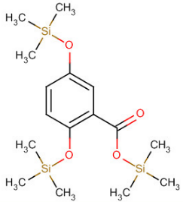 | 42.945 |
| 4575-74-0 | C_32_H_54_O_2_ | 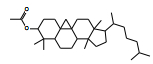 | 43.105 |
| 107098-30-6 | C_9_H_19_BCl_2_Si | 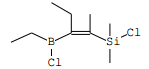 | 43.470 |
| 36638-45-6 | C_10_H_10_N_4_O_6_ | 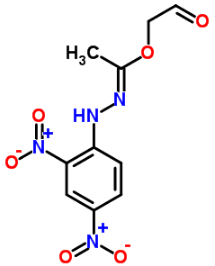 | 43.845 |
| 192461-49-7 | C_18_H_24_Si_2_ | 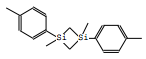 | 44.125 |
| 61000-04-2 | C_18_H_18_O_3_ | 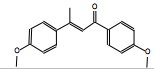 | 44.225 |
| 77636-36-3 | C_16_H_13_NO_4_ | 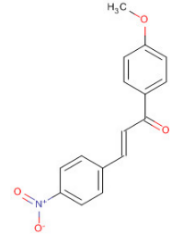 | 44.330 |
| 7604-99-1 | C_28_H_44_O_3_ | 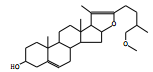 | 44.775 |
| 54889-97-3 | C_14_H_22_O_2_ | 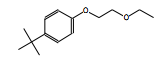 | 44.895 |
| 30262-98-7 | C_18_H_22_OSi | 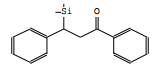 | 45.230 |
| 5508-47-4 | C_23_H_28_ClN_3_O_3_ | 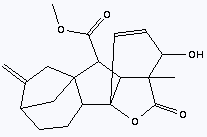 | 45.975 |
| 68002-20-0 | C_4_H_8_N_6_O | 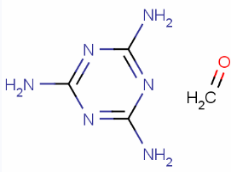 | 46.020 |
| 23066-93-5 | C_5_H_4_C_l2_O_3_ | 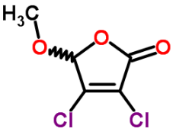 | 46.500 |
| 117-39-5 | C_15_H_10_O_6_ | 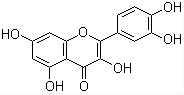 | 46.685 |
| 312532-08-4 | C_17_H_16_N_2_O_3_ | 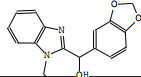 | 47.360 |

**Table S6** Concentrations of phenolic acids in root washings of Le and PI in a normal hydroponic culture system and under BYG stress (μg/L)

|  | normal hydroponic culture system | | | | | |  | under BYG stress | | | | | |
| --- | --- | --- | --- | --- | --- | --- | --- | --- | --- | --- | --- | --- | --- |
| 3-leaf | | 5-leaf | | 7-leaf | |  | 3-leaf | | 5-leaf | | 7-leaf | |  |
|  | Le | PI | Le | PI | Le | PI |  | Le | PI | Le | PI | Le | PI |
| 3, 4-Dihydroxybenzoic acid | 0b | 0.11±0.06a | 0.76±0.01a | 0b | 1.3±0.03a | 1.2±0.04b |  | 0 | 0 | 0.14±0.01a | 0b | 2±0.06a | 0.73±0.01b |
| *p*-Hydroxybenzoic acid | 1.1±0.06b | 6.3±0.07a | 3.4±0.03b | 4.9±0.09a | 0.35±0.01a | 0b |  | 0b | 5.1±0.11a | 3.9±0.16a | 3.7±0.03a | 2±0.08a | 0b |
| Vanillic acid | 1.09±0.01b | 3±0.01a | 2.22±0.04b | 5.13±0.05a | 1.47±0.03b | 4.96±0.08a |  | 0.13±0.01b | 1.87±0.11a | 1.11±0.02a | 0b | 2.43±0.06b | 3.51±0.06a |
| Syringic acid | 1.14±0.03a | 1±0.02b | 1.07±0.01b | 2.8±0.03a | 2.38±0.08a | 0.32±0.01b |  | 0b | 0.42±0.03a | 1.04±0.02b | 3.24±0.06a | 8.84±0.01a | 0.36±0.01b |
| Vanillin | 0.23±0.01b | 0.4±0.02a | 1.29±0.06b | 1.4±0.03a | 0.02±0.04a | 0.06±0.01a |  | 0 | 0 | 1.29±0.03a | 1.16±0.01b | 0 | 0 |
| Ferulic acid | 0 | 0 | 1.68±0.05a | 1.2±0.01b | 3.55±0.05a | 0.19±0.03b |  | 0b | 0.93±0.01a | 0.69±0.01a | 0.55±0.01b | 1.67±0.07a | 0b |
| Coumarin | 3.05±0.04a | 0.06±0.05b | 0.23±0.04b | 0.47±0.01a | 0.04±0.03b | 0.58±0.02a |  | 0.14±0.02a | 0b | 0.05±0.01a | 0b | 0 | 0 |
| Benzoic acid | 2.18±0.03b | 6.16±0.01a | 1.76±0.03b | 2.75±0.04a | 0b | 0.76±0.02a |  | 0b | 2.97±0.08a | 2.15±0.05b | 3.29±0.05a | 0.44±0.01b | 34.59±0.03a |
| Salicylic acid | 0 | 0 | 0 | 0 | 0 | 0 |  | 0 | 0 | 0 | 0 | 0 | 0 |
| Cinnamic acid | 0 | 0 | 0.14±0.16a | 0.32±0.01a | 0 | 0 |  | 0 | 0 | 0 | 0 | 0 | 0 |
| Sum | 8.8±0.03b | 17.03±0.01a | 12.55±0.04b | 18.98±0.02a | 9.11±0.01a | 8.07±0.01b |  | 0.27±0.03b | 11.28±0.07a | 10.37±0.05b | 11.94±0.01a | 17.39±0.03b | 39.19±0.02a |

Note: PI represents the wild–type allelopathic rice PI312227, Le represents the non-allelopathic rice Lemont. The data are expressed as the means ± SD of three replicates. Different lowercase letters indicate significant differences (LSD, p<0.05).

**Table S7** Concentration of total phenolics in root and leaf tissues measured using the Folin-Ciocalteu method (mg/g Root/Leaf fresh weight)

|  | Total concentration of phenolic compounds by sterilized water (mg/g) | | | | | | |
| --- | --- | --- | --- | --- | --- | --- | --- |
|  | Root | | | Rice accession | Leaf | | |
| Rice accession | 3-leaf | 5-leaf | 7-leaf |  | 3-leaf | 5-leaf | 7-leaf |
| PI | 0.1947±0.001b | 0.1697±0.0006b | 0.2373±0.0008b | PI | 0.5203±0.001a | 0.6418±0.0001b | 0.5763±0.0001b |
| PR | 0.1583±0.0013d | 0.1152±0.002d | 0.1098±0.002c | PR | 0.2546±0.003b | 0.5723±0.001d | 0.5188±0.001d |
| PO | 0.2730±0.08a | 0.1753±0.0007a | 0.2598±0.007a | PO | 0.5228±0.002a | 0.6559±0.0001a | 0.5868±0.0005a |
| Le | 0.1835±0.023c | 0.1566±0.01c | 0.2364±0.0001b | Le | 0.2536±0.0006b | 0.6375±0.0004c | 0.5504±0.0003c |
|  | Total concentration of phenolic compounds by methanol (mg/g) | | | | | | |
|  | 3-leaf | 5-leaf | 7-leaf |  | 3-leaf | 5-leaf | 7-leaf |
| PI | 0.5050±0.0001b | 0.4026±0.0005c | 0.2442±0.0007b | PI | 1.2473±0.0003b | 1.2114±0.0001b | 1.4634±0.0001b |
| PR | 0.2308±0.0007d | 0.1430±0.004d | 0.0974±0.0003d | PR | 0.8433±0.002c | 0.5015±0.0004d | 1.3087±0.001c |
| PO | 0.5286±0.002a | 0.6007±0.0004a | 0.5490±0.0009a | PO | 1.4531±0.0006a | 1.7846±0.0002a | 1.6250±0.006a |
| Le | 0.2368±0.001c | 0.4812±0.001b | 0.1498±0.002c | Le | 0.5531±0.0009d | 1.1107±0.001c | 0.8961±0.0006d |

Note: CK is the blank control, PI represents the wild–type allelopathic rice PI312227, PR represents *OsPAL2-1*-inhibited allelopathic rice, PO represents *OsPAL2-1*- overexpressed allelopathic rice, Le represents the non-allelopathic rice Lemont. The data are expressed as the means ± SD of three replicates. Different lowercase letters indicate significant differences (LSD, p<0.05).

**Figure**


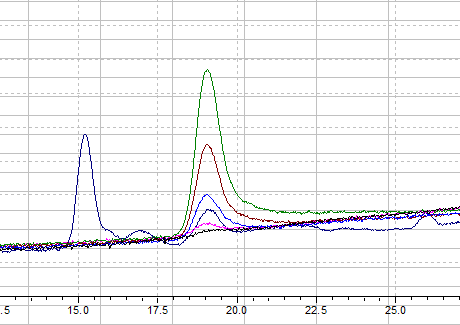


3.31×10^-4^mmol/L

6.62×10^-4^mmol/L

0.33×10^-2^mmol/L

0.66×10^-2^mmol/L

0.17×10^-2^mmol/L

PA_1_

**Figure S1** HPLC of quercetin and the sample


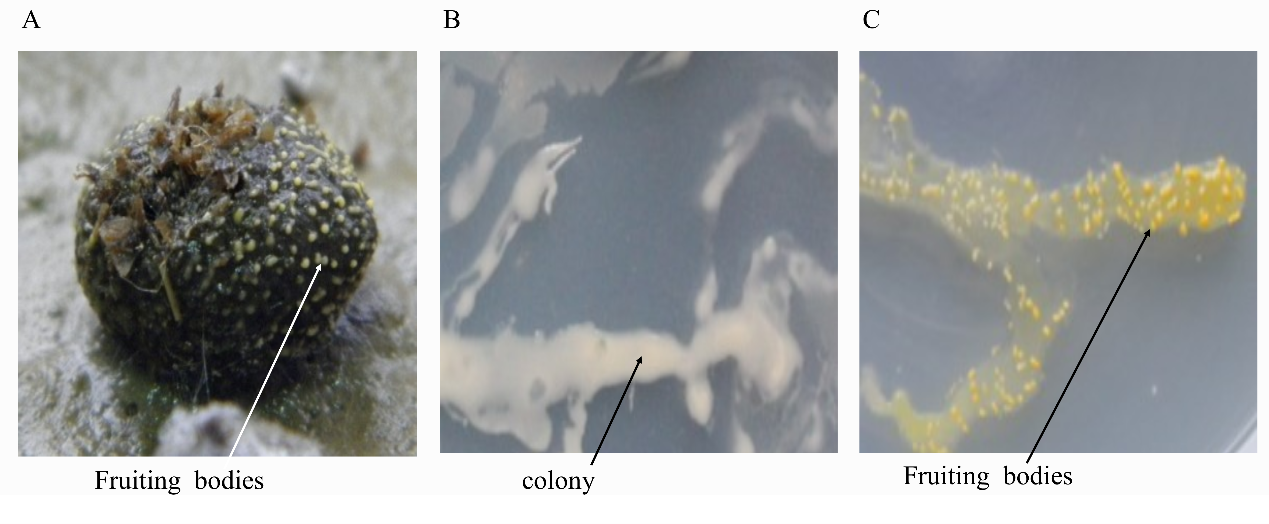


**Figure S2** The morphology of fruiting bodies (A) of the strain induced by rabbit feces and the morphology of fruiting bodies (B) and the colony (C) of the purified strain
